# Good for Mexico

**Published:** 1883-09

**Authors:** 


### GOOD FOR MEXICO.

Dr. D. F. Patino, in an article published in *La Escuela de Medicina* of Mexico, tells us of a fact which is highly creditable to our neighbor republic. Dr. Patino asserts and proves that Mexico was the first country in the world which founded an Insane Asylum. In the XVI century, during the Spanish domination, a lay-friar of the Convent of St. Francisco de Mexico, a brother named Bernardino Alvarez, conceived the idea or plan of isolating the poor insane in a house which he fitted up with his own means and the help received from public charity. Thus, the first insane asylum was founded and located in a street of the City of Mexico, called then De la Celado, at present St. Bernard Street, almost in the same place where to-day stands the church of that name. Till then, that is till the XVI century and during the first periods of Spanish Domination, the insane could be seen wandering along the streets, followed and persecuted by crowds of vagabonds and mischievious boys, who continually tormented them. The furiously-mad were immured in little cells having only a small window in the roof through which was thrown to them their food, which was rarely touched, as their lives were being extinguished by degrees amid the most inconceivable sufferings.

Brother Bernardino Alvarez conceived the idea of collecting those poor creatures, that science and charity might bring them again to life, to reason, to feeling and intelligence.—*El Progresso Dental*, Havana.

